# Self-Powered Flow Rate Sensing via a Single-Electrode Flowing Liquid Based Triboelectric Nanogenerator

**DOI:** 10.3390/mi15030384

**Published:** 2024-03-13

**Authors:** Duy-Linh Vu, Quang-Tan Nguyen, Pil-Seung Chung, Kyoung-Kwan Ahn

**Affiliations:** 1 Department of Nanoscience and Engineering, Inje University, 197 Inje-ro, Gimhae-si 50834, Gyeongsangnamdo, Republic of Korea; vuduylinhbk@gmail.com; 2 School of Mechanical Engineering, University of Ulsan, 93 Daehak-ro, Nam-gu, Ulsan 44610, Republic of Korea; pax.quangtan@gmail.com; 3 Department of Energy Engineering, Inje University, 197 Inje-ro, Gimhae-si 50834, Gyeongsangnamdo, Republic of Korea

**Keywords:** self-powered sensing, flow rate, silicon pipe, coated electrode, triboelectric nanogenerator

## Abstract

Recently, triboelectric nanogenerators (TENGs) have emerged as having an important role in the next wave of technology due to their large potential applications in energy harvesting and smart sensing. Recognizing this, a device based on TENGs, which can solve some of the problems in the liquid flow measurement process, was considered. In this paper, a new method to measure the liquid flow rate through a pipe which is based on the triboelectric effect is reported. A single-electrode flowing liquid-based TENG (FL-TENG) was developed, comprising a silicon pipe and an electrode coated with a polyvinylidene fluoride (PVDF) membrane. The measured electrical responses show that the FL-TENG can generate a peak open-circuit voltage and peak short-circuit current of 2.6 V and 0.3 µA when DI water is passed through an 8 mm cell FL-TENG at a flow rate of 130 mL/min and reach their maximum values of 17.8 V–1.57 µA at a flow rate of 1170 mL/min, respectively. Importantly, the FL-TENG demonstrates a robust linear correlation between its electrical output and the flow rate, with the correlation coefficient R^2^ ranging from 0.943 to 0.996. Additionally, this study explores the potential of the FL-TENG to serve as a self-powered sensor power supply in future applications, emphasizing its adaptability as both a flow rate sensor and an energy harvesting device.

## 1. Introduction

Given the rapid advancement of internet technology and the growing needs of individuals, there is an urgent need for the establishment of new, trustworthy, and sustainable operations for sensor networks. The advancement of sensing networks has played a pivotal role in shaping the trajectory of future developments. Sensors have progressively become integral to various facets of daily life, contributing significantly to enhancing processes such as energy harvesting, promoting health and safety, ensuring security, and facilitating environmental monitoring [1,2,3,4]. Over the past few decades, a number of sensor types have been innovated to measure diverse physical factors, including temperature, humidity, pressure, motion, vibration, and gas concentration [5,6,7]. Earth experiences frequent occurrences of water disasters, which emphasizes the importance of setting up a real-time fluid flow monitoring system. It is imperative to employ electronic strategies for gauging the real-time velocity of fluid flow, particularly in challenging environments, as opposed to relying on direct detection [8,9,10]. Current technologies for flow rate measurements include assessing the rotational frequency of a turbine in a turbine flow meter, evaluating the pressure drop across an orifice flow meter, and measuring the electromotive force in an electromagnetic flowmeter [11,12,13]. Additionally, the transit time or Doppler effect is utilized in ultrasonic flow meters [14,15]. However, each of these technologies has inherent drawbacks, such as fluid flow disturbances, pressure drops, high costs, and limitations in applications with millimeter-scale pipes [16,17]. Furthermore, these technologies necessitate a continuous external power supply and ample internal space for integration into the device. On the other hand, self-powered sensing involves sensors that can autonomously harvest energy from sources like light, heat, or mechanical energy. They convert this energy into an electrical signal in response to ambient environmental stimuli, eliminating the need for any external power source to be applied to the device [18,19,20]. Consequently, there is considerable desirability to develop self-powered sensors for flow rate detection, characterized by high accuracy, stability, and an extended operational lifetime.

Over the past decade, nanotechnology has undergone rapid advancements, leading to the development and utilization of numerous nanogenerators in energy harvesting systems and self-powered smart devices. Since the introduction of the triboelectric nanogenerator (TENG) in 2012, various research groups have created and shared applications of TENGs [21,22,23,24,25,26]. These generators demonstrate significant potential for effective energy harvesting and self-powered sensors. By combining triboelectrification and electrostatic induction, these sensors have the ability to directly transform irregular and small-scale mechanical energy into an electric signal without the need for external power. This characteristic makes them well-suited for establishing a comprehensive real-time sensor network on a large scale [27,28,29]. Nevertheless, there are several crucial issues that need attention. Firstly, it is essential to create a motion sensor with high accuracy and a wide detection range. Secondly, the friction caused by sliding modes can result in mechanical wear of the dielectric materials, presenting a significant challenge to the long life of the sensor [30,31,32]. Additionally, a waterproof device is indispensable to maintain a high triboelectrification effect in outdoor environments, particularly in fluid conditions. Fortunately, triboelectric devices are not limited to solid–solid materials; they can also involve liquid–solid materials. Leveraging liquid–solid contact electrification has enabled the realization of various applications for harvesting water energy [33,34,35,36,37,38].

Previous studies indicate that triboelectricity can be generated through various types of friction between liquid and solid materials, including water flowing through a pipe [39,40,41,42]. Specifically, when a triboelectric layer becomes wetted by a liquid, the electrical double layer (EDL) at the liquid triboelectric layer interface screens the electrostatic induction of the back electrode, resulting in a measured charge [34,43]. Consequently, this generated signal can serve as an indicator of the wetting state of a flow rate pipeline. However, the comprehensive understanding of flow rate factors such as flow state, pipe diameter, and ion concentration in the liquid has not been extensively explored. Therefore, this paper introduces a single-electrode flowing liquid-based triboelectric nanogenerator (FL-TENG) as a novel approach for liquid flow rate measurement. The proposed method offers advantages such as low cost, easy installation, high sensitivity, and suitability for millimeter-scale pipes. Experimental validations were conducted using three different millimeter-scale pipes with inner diameters of 3, 5, and 8 mm. The liquids used in the research included DI water, tap water, and seawater. A commercially available PVDF thin film served as the negatively charged triboelectric layer. The generated power of the FL-TENG was measured and analyzed to establish the relationship between the electrical response and liquid flow rate through the FL-TENG device. From the results, the FL-TENG demonstrates a robust linear correlation between the produced electrical output and the flow rate, with the correlation coefficient R^2^ ranging from 0.943 to 0.996. Furthermore, the performance generated potential of the FL-TENG to function as a self-powered sensor power supply in future developments was also considered. This study underscores the potential utility of FL-TENGs as both a flow rate sensor and an energy harvester.

## 2. Experimental Section

### 2.1. Fabricaiton of FL-TENG Cells

**Figure 1:**
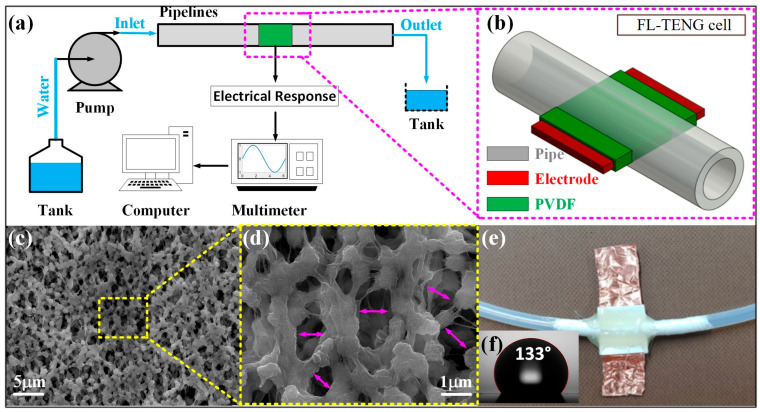
(**a**) Schematic diagram of FL-TENG experiment setup, (**b**) illustration of FL-TENG cell, (**c**,**d**) SEM image of PVDF membrane and its zoomed-in image, with double arrows, depicts the pore dimensions, (**e**) real photograph of FL-TENG, (**f**) contact angle PVDF membrane.

An FL-TENG cell comprises three components: a silicon pipe, a copper electrode serving as the charge-collecting layer, and a PVDF membrane functioning as the charge-generating layer (as shown in Figure 1). The fabrication of the cell is uncomplicated, involving the creation of a triboelectric layer by covering a copper electrode (width: 10 mm, thickness: 100 µm) with a PVDF membrane (thickness: 50 µm). The triboelectric layer extends through the wall of a silicon pipe with inner diameter sizes of 3, 5, and 8 mm. These different sizes were used to distinguish between various FL-TENGs in subsequent discussions, for instance, a 3 mm cell. The cell is connected to the ground via an external circuit, resulting in a single-electrode mode for the TENG. The schematic and real images of the FL-TENG cell are depicted in Figure 1b,e.

Moreover, the surface morphology of the PVDF membrane (Sigma-Aldrich, St. Louis, MO, USA) was examined through FE-SEM by using JSM-7600 FE-SEM from JEOL (Tokyo, Japan) and presented at different scales in Figure 1c,d, revealing a highly porous surface. The pores are evenly distributed on the membrane surface, with sizes ranging from around 500 nm to 800 nm. The porous structure assumes significance in liquid–solid contact due to the highly hydrophobic surface and specific contact area. Figure 1f illustrates the contact angle between a 10 µL water droplet and the PVDF surface through SmartDrop, FemtoFAB (Waltham, MA, USA). The water contact angle of the PVDF membrane reaches 133°, indicating a highly hydrophobic surface. Consequently, water can easily slip on the PVDF surface, enhancing the contact–separation at the interface and thereby improving the output performance of the TENG.

### 2.2. Structural Analysis and Design Techniques

The experiment setup for the FL-TENG is illustrated in Figure 1a. The structured analysis and design techniques method was employed to delineate the function of the proposed FL-TENG system. In this system, the flowing liquid serves as the input, and the generated electricity serves as the output. The process function relies on triboelectric charging occurring when the liquid comes into contact with a triboelectric layer. The electrical responses are significantly influenced by the liquid flow rate, the dimensions of the silicon pipe, and the properties of the liquids, making these factors essential considerations as control activities in the process.

To quantify the generated output, a digital graphical sampling multimeter Keithley DMM7510 (Keithley Instruments, Solon, OH, USA) was utilized. The open-circuit voltage and short-circuit current were measured to assess the performance of the FL-TENG cell. The AC-generated voltage was rectified to DC using a bridge rectifier. The output power was stored in capacitors with varying capacitance values. A peristaltic pump was employed to generate liquid flow, and the liquid flow rate was directly controlled by configuring the pump parameters. The peristaltic pump consisted of a rotor with several rollers (in this case, a three-roller pump) attached to an external flexible tube. As the rotor turned, the compression of the tube closed a portion, forcing the fluid to be pumped through the pipe.

## 3. Results and Discussion

### 3.1. Working Mechanisms of the FL-TENG

**Figure 2:**
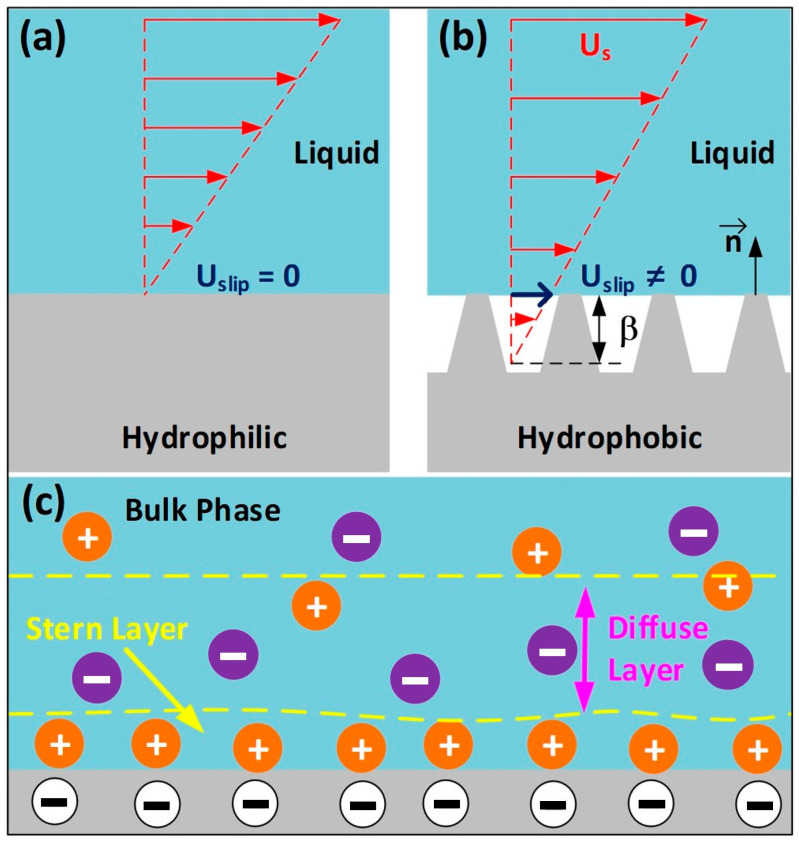
(**a**,**b**) Schematic diagram of slip conditions at the liquid-solid interfaces; (**c**) charge distribution at the liquid-solid interfaces.

The operational mechanism of the FL-TENG was established on the principles of liquid–solid contact electrification, involving the interaction between a liquid serving as a positively charged material and the PVDF membrane serving as negatively charged. Traditionally, most previous studies addressing flowing liquid scenarios have adopted the no-slip condition, assuming the immobility of ions at the liquid–solid interface. However, advancements in micro- and nanotechnologies enable the modification of liquid–solid interface properties, challenging the accuracy of the no-slip condition [44]. Therefore, it becomes imperative to consider the hydrodynamic slip condition, as depicted in Figure 2. The characteristics of liquid–solid interfaces can be altered at microscopic and nanoscopic scales, affecting parameters like wettability and roughness [45]. Following the Navier–slip boundary condition, the slip velocity can be calculated [46]:

(1) $$U_{slip}=\beta \left(\partial U_{s}/\partial n\right)$$

where $U_{slip}$ is the slip velocity, β is the slip length, $U_{s}$ is the liquid velocity parallel to the surface, and n is the unit vector normal to the solid surface. A schematic representation of the slip condition at the liquid–solid interfaces is illustrated in Figure 2a,b. Recent experiments demonstrate that water adheres without slipping on a hydrophilic surface but exhibits slip on a hydrophobic surface. The rough structure of a hydrophobic surface can increase the slip length [47]. The slip boundary condition is contingent on the shear rate near the surface, and over a hydrophobic surface, the slip length increases linearly with the shear rate [48]. In a pipe, the velocity increases as one moves toward the center, generating shear forces due to differential velocities between adjacent liquid layers. Furthermore, the charge distribution at the liquid–solid interface is depicted in Figure 2c. The FL-TENG comprises a copper electrode enveloped by a PVDF membrane attached to the pipe. Upon contact with the liquid, the PVDF surface becomes negatively charged. To neutralize this charged PVDF surface, an EDL is formed, with cations adsorbed onto the negative surface of the FL-TENG and counter-ions residing in the diffuse layer [49].

The movement of adsorbed ions on the PVDF surface can be influenced by an externally applied force, aligning with the direction of the force. As the liquid flows through the pipe, these adsorbed ions travel along with the liquid, creating a force on the ions that intensifies with the liquid flow. The displaced adsorbed ions generate a voltage in the FL-TENG through electronic friction with free electrons within the PVDF triboelectric layer. In the static state, there is a net influx of ions adsorbed onto the FL-TENG upstream and a net efflux of ions desorbed downstream. The flowing liquid carries cations away from the EDL, introducing more charges across the liquid–solid interfaces to replenish the EDL. Electrons then alternately flow between the PVDF triboelectric layer and the ground through the external circuit, resulting in an AC-type current output.

**Figure 3:**
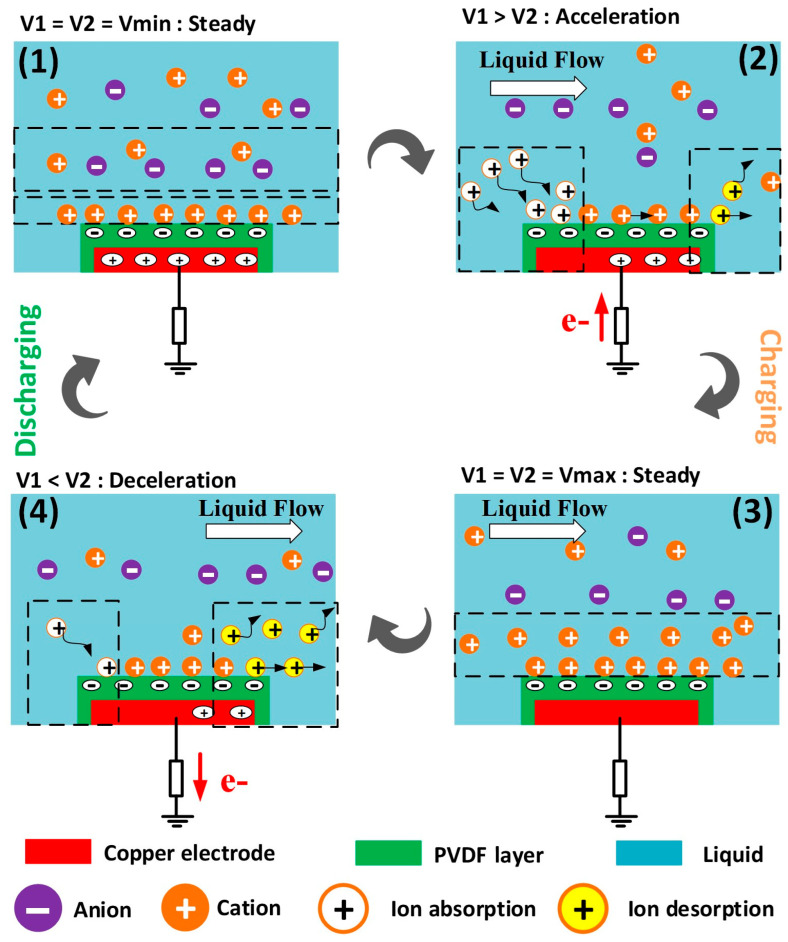
Schematic working principle of the FL-TENG.

The liquid flow rate is controlled using a peristaltic pump based on tube compression/relaxation. During the relaxation and compression phases, the liquid flow rate at the pump outlet or the rate of liquid coming into contact with the FL-TENG may be accelerated or decelerated, respectively. Figure 3 illustrates the operating principle of the FL-TENG: (1) initial state—the liquid is steady and the EDL is established, creating a potential difference at the electrode; (2) acceleration—the liquid is accelerated through the pipe during the relaxation phase of the pump. On the left side (upstream) of the PVDF membrane, cations in the flowing liquid are adsorbed onto the PVDF surface, while on the right side (downstream), the adsorbed cations move away from the PVDF membrane. In the case of acceleration, the adsorbed ions on the left side exceed the desorbed ions, resulting in a surplus of positive charges at the interface. To replenish the EDL, electrons must flow from the ground, leading to current generation. This process is known as charging; (3) steady state—when the liquid flow is constant, the EDL is balanced; (4) deceleration—the flow rate decreases during the compression phase of the pump. In this scenario, the ion adsorption is less than desorption, and electrons flow back to the ground, generating electricity. This charging/discharging process repeats continuously.

### 3.2. Electrical Output Characteristics of the FL-TENG

**Figure 4:**
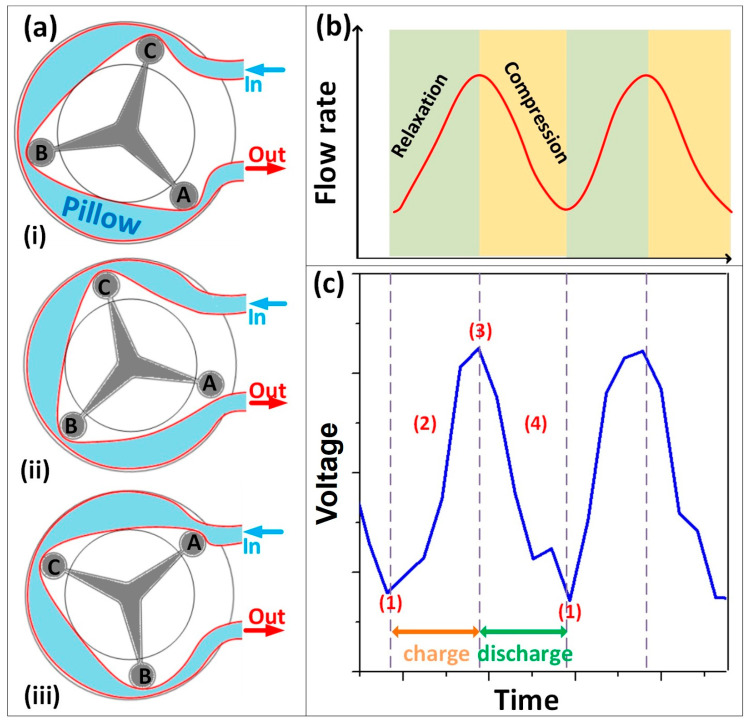
(**a**(**i**)–(**iii**)) Working principle of peristaltic pump in different roller position; (**b**) schematic diagram of the flow rate versus time of a peristaltic pump; and (**c**) the corresponding generated electrical signal of FL-TENG to the four states of working principle represented by (1)–(4).

To induce water movement in the pipe, a three-roller peristaltic pump (Ecoline VC-360, Fisher Scientific, Pittsburgh, PA, USA) was used. The pump contains a flexible pipe fitted inside its casing, positioned between the tube bed and the rotor, as illustrated in Figure 4a. At locations A, B, and C, the pipe is compressed by each roller, creating a liquid pillow between two rollers. The pumping mechanism relies on the periodic compression and relaxation of the tube, drawing content in and propelling product away from the pump. As roller A moves up during the relaxation process, the flow rate at the outlet accelerates (Figure 4a(ii)). When the compression process occurs with roller B, the outlet flow rate decelerates (Figure 4a(iii)), continuing until roller B returns to its initial position relative to roller A, completing a full relaxation/compression cycle. This process repeats, resulting in an alternating acceleration and deceleration liquid flow rate profile, as depicted in Figure 4b. The liquid is then directed to make contact with the FL-TENG, initiating triboelectrification to generate an electrical signal. The electrical response aligns with the liquid flow rate profile, as shown in Figure 4b,c.

The volume flow rate is defined as

(2) Q=A×v

where *Q* is the liquid flow rate, *A* is the cross-sectional area normal to the flow direction, and v is the average liquid velocity normal to the cross-sectional area. The cross-sectional area is calculated as follows:

(3) $$A=0.25\pi d^{2}$$

where *d* is the diameter of the flexible tube. Therefore, the relationship between the average velocity and the rotation speed is given by

(4) $$Q=0.5\pi^{2}d^{2}Nr$$

where *N* is the speed of rotation (rpm) and *r* is the radius of rotation measured from the rotor axis to the center of the rollers. The relationship between the rotation speed and flow rate is displayed in Figure S1 (Supplementary Materials) for the peristaltic pump calculation using an 8 mm cell FL-TENG.

**Figure 5:**
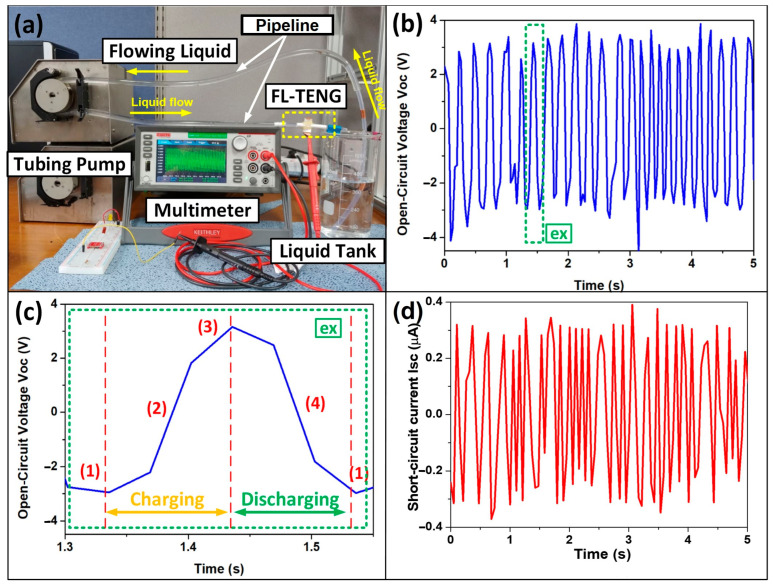
Output performance of the FL-TENG with a flowing DI water of 390 mL/min, using an 8 mm cell device. (**a**) Real photograph of the FL-TENG testbench; (**b**) open-circuit voltage (*V_oc_*) and (**c**) enlarged *V_oc_* for the correspondence between the four state ((1)–(4)) of flowing liquid through the FL-TENG and its output values; and (**d**) short-circuit current (*I_sc_*).

To showcase the energy-generating capabilities of the FL-TENG, we conducted measurements on the electrical output characteristics while water flowed through an 8 mm cell device, and deionized (DI) water was employed. In this investigation, the pump operated at a speed of 105 rpm, corresponding to a water flow rate of 390 mL/min. The experimental setup for the FL-TENG is depicted in Figure 5a. As illustrated in Figure 5b,d, the peaks of the open-circuit voltage (*V_oc_*) and short-circuit current (*I_sc_*) exhibit high uniformity, making these data advantageous for sensing signals. The peak-to-peak of the *V_oc_* and *I_sc_* is also significantly high, measuring 7.2 V and 0.59 µA, respectively. This indicates a substantial potential for generating sufficient energy to power the sensor device, and further details will be presented in the next section. Moreover, Figure 5c provides an illustration of the charging/discharging process of the FL-TENG. The observed output signal serves as evidence that electricity can be generated when the liquid comes into contact with the FL-TENG device, positioned in the middle of the pipe, due to triboelectrification. In practical applications, a sensing device needs to remain stable and durable for accurate measurements. The experiments were conducted a month apart to verify the stability of the FL-TENG. As depicted in Figure S2 (Supplementary Material), the *V_oc_* of the FL-TENG shows minimal changes in its electrical performance at water flow rates of 390 mL/min and 650 mL/min, indicating strong durability and stability during operation.

### 3.3. FL-TENG as a Liquid Flow Rate Sensor

**Figure 6:**
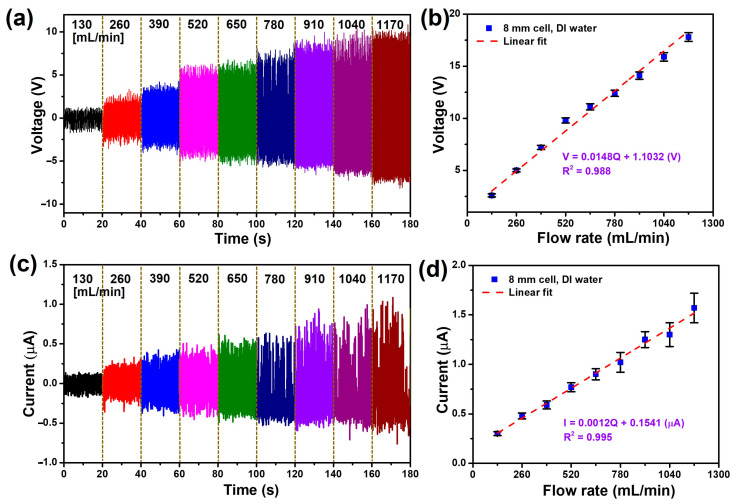
(**a**) Output voltage versus time with different flow rates and (**b**) linear relationship between the output voltage and flow rate; (**c**) output current versus time with different flow rates and (**d**) linear relationship between the output current and flow rate.

To assess the potential of the FL-TENG as a flow rate sensor, an examination of the relationship between the generated electricity and various flow rates ranging from 130 to 1170 cc/min is imperative. The experiment continued to employ the FL-TENG featuring an 8 mm cell device and deionized (DI) water. Illustrated in Figure 6a,c, at a flow rate of 130 mL/min, the FL-TENG yields peak-to-peak values of the *V_oc_* and *I_sc_* measuring 2.5 V and 0.3 µA, respectively. These values escalate to 11.1 V and 0.9 µA at a flow rate of 650 mL/min and reach their maximum values at 17.8 V–1.57 µA at a flow rate of 1170 mL/min, respectively. These observed enhancements can be attributed to the heightened velocity of water flowing through the TENG device, which imparts a more substantial impact force on the surface of the PVDF membrane. This intensified impact force contributes to the expansion of the EDL and the generation of additional surface charges [50]. Consequently, the interaction dynamics between the flowing water and the triboelectric surface, both upon relaxation/compression cycles, undergo acceleration. This acceleration in the interaction process results in a notable increase in both the intensity and frequency of the output current and voltage peaks.

As evident in Figure 6b,d, both the *V_oc_* and *I_sc_* exhibit an upward trend with the flow rate. Linear fittings of the measured voltages/current against the flow rate reveal high correlation coefficients, specifically R^2^ = 0.988 and 0.995, respectively. The regression curves for these relationships are, respectively, delineated by

(5) $$V_{oc}=0.0148Q+1.1032\left(V\right)$$

(6) $$I_{sc}=0.0012Q+0.1541\left(\mu A\right)$$

where *Q* is the liquid flow rate (mL/min). Consequently, the *V_oc_* and *I_sc_* generated by the liquid flow through the FL-TENG demonstrate high sensitivities of approximately 0.0148 V/mL·min and 0.0012 µA/mL·min, respectively. Given the robust linear relationship between the generated electricity and liquid flow rate, the FL-TENG exhibits promising potential for development as a flow rate sensor. However, as the water flow rate increases to 780 mL/min, the peaks of *I_sc_* exhibit irregularities, potentially introducing errors in the calculation of flow rates. Hence, in the subsequent investigation, our emphasis is on examining the correlation between the output voltage and flow rate under varying conditions.

**Figure 7:**
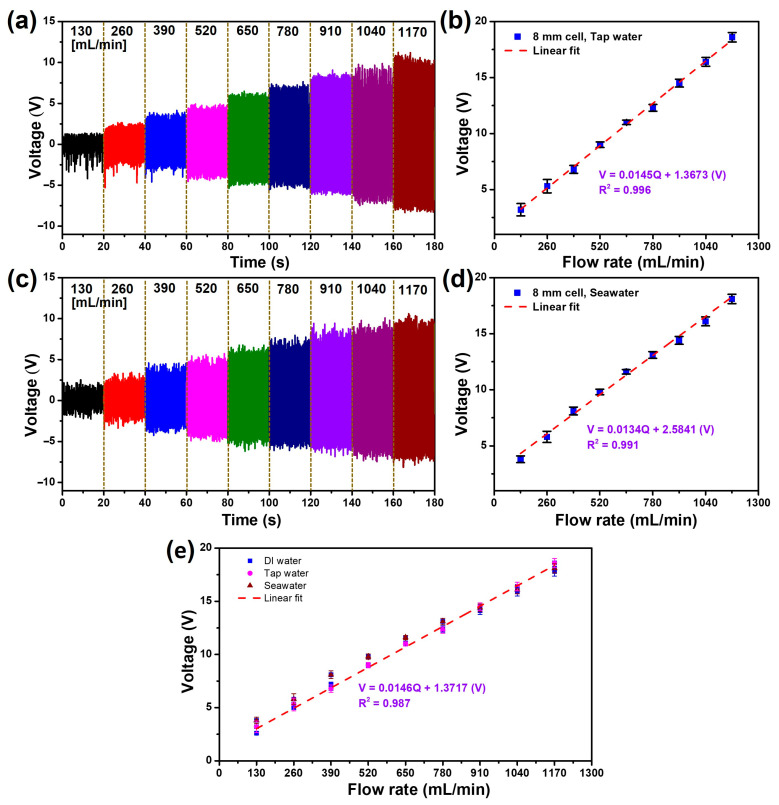
Output voltages with different flow rates and their corresponding linear relationship between output voltage and average flow rate when using (**a**,**b**) tap water and (**c**,**d**) seawater; (**e**) the linear regression line for three different liquids with flow rates.

Figure 7 illustrates the output voltage and the correlation between the electrical response and liquid flow rate when employing tap water and seawater as alternatives to DI water. For this study, tap water was procured from a commercial provider (K-water, Ulsan, Republic of Korea), characterized by moderate mineralization featuring 200–700 mg/L concentrations of Na^+^, Mg^2+^, and Ca^2+^ ions, along with trace amounts of F^−^, Cu^2+^, Zn^2+^, and K^+^ ions. Seawater was sourced from Ulsan Bay and comprised approximately 96.5% water and 2.5% salts, containing small minor quantities of ions such as Mg^2+^, Ca^2+^, K^+^, and SO_4_^2−^. The experiments were conducted using an FL-TENG with an 8 mm cell device, examining the relationship between the voltage signal and liquid flow rates ranging from 130 mL/min to 1170 mL/min. As depicted in Figure 7a,c, the generated *V_oc_* increases proportionally with the rise in flow rates, following the same trend observed with DI water. Specifically, at a flow rate of 130 mL/min, the FL-TENG produces a peak-to-peak *V_oc_* of 3.2 V and 3.5 V, then reaches maximum values of 18.6 V and 18.1 V at 1170 mL/min when employing tap water and seawater, respectively. As observed, the output voltage exhibits a slight increase when tap water and seawater are used instead of DI water. This phenomenon can be attributed to the presence of ions in tap water and seawater, which impart a level of conductivity conducive to the conversion between the built-in electric fields at the interface. This increased conductivity facilitates the more efficient transfer of electrons from the sliding surface of water, consequently resulting in an elevation of the output signal in the FL-TENG device [51].

Furthermore, in Figure 7c,d, the correlation between the measured voltages and flow rates is effectively illustrated through linear fittings, demonstrating high correlation coefficients (R^2^ values of 0.995 and 0.991). Specifically, when utilizing tap water, a linear relationship is established with a sensitivity of 0.0145 V/mL·min, and with seawater, a sensitivity of 0.0134 V/mL·min is achieved, close to the sensitivity observed with DI water. As a result, an attempt was made to integrate all three types of liquid to formulate a generalized equation capable of measuring various solutions irrespective of their ion content (Figure 7e). The regression curve for this relationship is outlined as follows:

(7) $$V=0.0146Q+1.3717\left(V\right)$$

Consequently, for the peak-to-peak voltage signal measured across different flow rates under three liquid types, a sensitivity of 0.0146 V/mL·min is derived with a high coefficient of determination (R^2^ value of 0.987). These elevated sensitivities and high coefficients of determination affirm the great accuracy of the measurement, enabling the estimation of flow rates through the monitoring of the output voltage signal of the FL-TENG device. This observation underscores the negligible influence of the test liquid on the output voltage of the FL-TENG, affirming the stability of the sensing signal.

**Figure 8:**
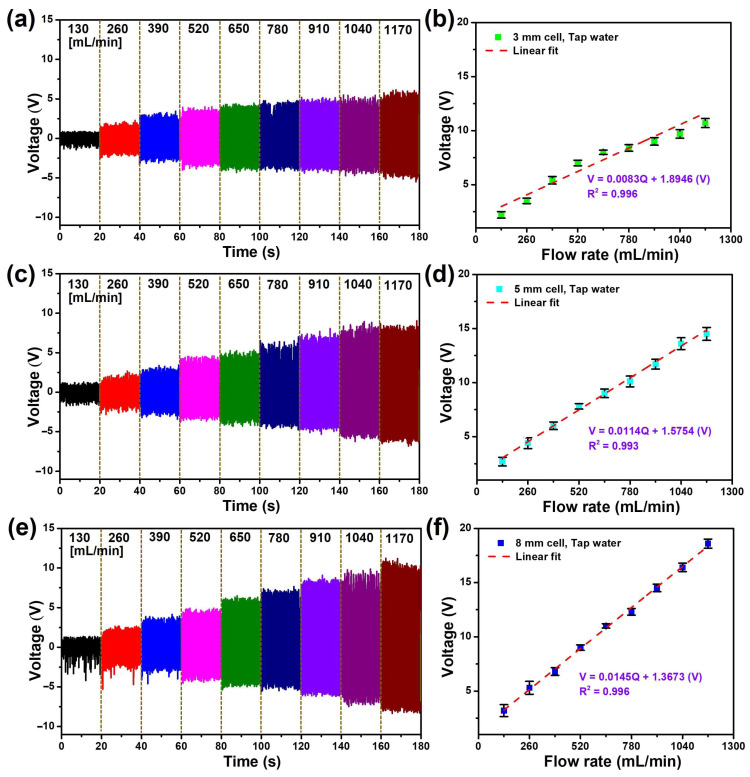
Output voltage at various tap water flow rates, along with the respective linear relationships between the voltage and flow rate when using (**a**,**b**) 3 mm cell; (**c**,**d**) 5 mm cell; (**e**,**f**) 8 mm cell FL-TENG devices.

On the other hand, to showcase the accuracy of the FL-TENG in millimeter-scale measurements, various cell sizes, including 3 mm cell, 5 mm cell, and 8 mm cell were examined. Figure 8a,c,e present the output voltage at various flow rates of tap water ranging from 130 to 1170 mL/min. The peak-to-peak voltage of the 3 mm cell and 5 mm cell FL-TENGs follows the same pattern as the 8 mm cell discussed in the preceding section. However, concerning flow rates, the voltage demonstrates an increase with the rise in pipe diameter. This phenomenon could be attributed to the larger diameter, resulting in a higher contact surface of the FL-TENG device and, consequently, an increase in electrical charge. Therefore, an increase in the output voltage was confirmed. Consequently, sensitivities of 0.0083, 0.0114, and 0.0145 V/mL·min were determined for the 3 mm cell, 5 mm cell, and 8 mm cell, respectively, corresponding to R^2^ values of 0.943, 0.993, and 0.996 (Figure 8b,d,f). Despite the 3 mm cell exhibiting a significantly smaller sensitivity compared to the other two FL-TENGs, it still exhibits a good linear relationship, affirming the suitability of the FL-TENG for accurate millimeter-scale flow rate measurement. These discussions demonstrate that monitoring the voltage signals of the FL-TENG enables the determination of the water flow rate.

### 3.4. FL-TENG as an Energy Harvester

**Figure 9:**
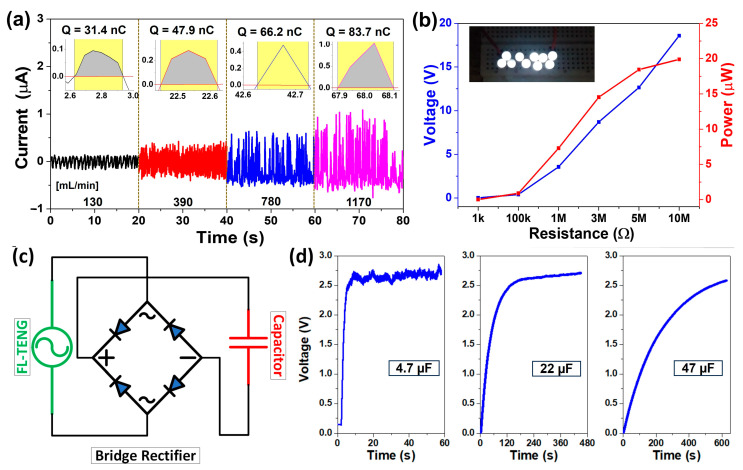
Demonstration of the FL-TENG as an energy harvesting system. (**a**) Short-circuit currents and their corresponding calculation of a single peak transfer charge at various flow rates; (**b**) voltage and peak power of the FL-TENG across different resistance values; (**c**) schematic representation of the bridge rectifier circuit; and (**d**) voltage of the storage capacitor over time, showcasing variations with different capacitance values (4.7 µF, 22 µF, and 47 µF).

As a self-powered sensor device, the output performance of the FL-TENG also attracted a lot of attention to prove it has enough power to supply sensor devices. In Figure 9, we demonstrate the ability of the FL-TENG as an energy harvesting system. The FL-TENG device was operated using tap water, an 8 mm cell, and an 1170 mL/min flow rate. Figure 9a shows short-circuit currents and their corresponding calculation of a single peak transfer charge at various flow rates. The current is related to the rate of charge transfer:

(8) $$I_{sc}=\frac{{dQ}_{sc}}{dt}$$

where *I_sc_* and *Q_sc_* represent short-circuit current and transferred charges, respectively. As depicted, the FL-TENG exhibits a charge transfer of 31.4 nC at a flow rate of 130 mL/min, reaching a maximum of approximately 83.7 nC at 1170 mL/min. Furthermore, Figure 9b illustrates the voltage and peak power of the FL-TENG across various resistance values (ranging from 1 kΩ to 10 MΩ). The instantaneous power was calculated and peaked at 19.9 µW at a flow rate of 1170 mL/min. Notably, this power is adequate to directly illuminate a series of 10 white LEDs, as depicted in the inset figures. To store the generated power, a bridge rectifier was connected to the FL-TENG, converting the electrical output from AC-type to DC-type and subsequently charging capacitors, as shown in Figure 9c. Different capacitors were employed to assess the performance of the device under varying capacitive loads. In Figure 9d, it is demonstrated that to attain a 2.5 V DC output, the FL-TENG can charge a 4.7 µF capacitor within 10 s. In the case of 22 µF and 47 µF capacitors, the required charging times are 120 s and 600 s, respectively. This study underscores the potential of the FL-TENG as an energy source for self-powered device applications.

## 4. Conclusions

This study introduces a novel approach to flow rate measurement utilizing liquid–solid contact triboelectrification. The recorded electrical response demonstrates a gradual increase in the peak-to-peak open-circuit voltage and short-circuit current of the FL-TENG device with a rise in the liquid flow rate. Calculations indicate that the instantaneous power peaks at 19.9 µW at a flow rate of 1170 mL/min, proving sufficient to directly illuminate a series of 10 white LEDs. This finding underscores the potential for energy supply from the FL-TENG device to power sensor devices effectively. The FL-TENG exhibits a highly linear relationship between the voltage response and liquid flow rate, yielding a sensitivity of 0.0146 V/mL·min and a high coefficient of determination (R^2^ value of 0.987), unaffected by ions in the solution. Furthermore, investigations into the size of the TENG, involving various cell sizes (3 mm cell, 5 mm cell, and 8 mm cell), reveal sensitivities of 0.0083, 0.0114, and 0.0145 V/mL·min for the respective cell sizes, with corresponding R^2^ values of 0.943, 0.993, and 0.996. The proposed flow rate measurement method offers advantages such as cost-effectiveness, easy installation, and stability. These studies, emphasizing sensitivity and energy harvesting potential, underscore the viability of the FL-TENG as a liquid flow rate self-powered sensing device and to harvest energy from flowing liquids.

## Data Availability

Data are contained within the article.

## Supplementary Materials

The following supporting information can be downloaded at: https://www.mdpi.com/article/10.3390/mi15030384/s1, Figure S1: Characteristics of the peristaltic pump with 8 mm cell FL-TENG; Figure S2: Stability and durability of the FL-TENG: output voltage measured at (a) 390 and (b) 650 mL/min of the FL-TENG at two moments one month apart.
