# Supplementary material for: Self-Powered Flow Rate Sensing via a Single-Electrode Flowing Liquid Based Triboelectric Nanogenerator
Source: Micromachines (Basel). 2024 Mar 13;15(3):384. doi: 10.3390/mi15030384 (PMC10972509; doi:10.3390/mi15030384)
Supplement: Supplementary file 1 [file micromachines-15-00384-s001.zip › micromachines-2873890-supplementary.pdf]

micromachines

MDPI

Supplementary Materials

Self-Powered Flow Rate Sensing via a Single-Electrode Flowing Liquid Based Triboelectric Nanogenerator

Duy-Linh Vu [1], Quang-Tan Nguyen [2], Pil-Seung Chung [1,3,*] and Kyoung-Kwan Ahn [2,*]

[1] Department of Nanoscience and Engineering, Inje University, 197 Inje-ro, Gimhae-si 50834, Gyeongsangnamdo, Republic of Korea; vuduylinhbk@gmail.com
[2] School of Mechanical Engineering, University of Ulsan, 93 Daehak-ro, Nam-gu, Ulsan 44610, Republic of Korea; pax.quangtan@gmail.com
[3] Department of Energy Engineering, Inje University, 197 Inje-ro, Gimhae-si 50834, Gyeongsangnamdo, Republic of Korea
* Correspondence: pschung01@inje.ac.kr (P.-S.C.); kkahn@ulsan.ac.kr (K.-K.A.)

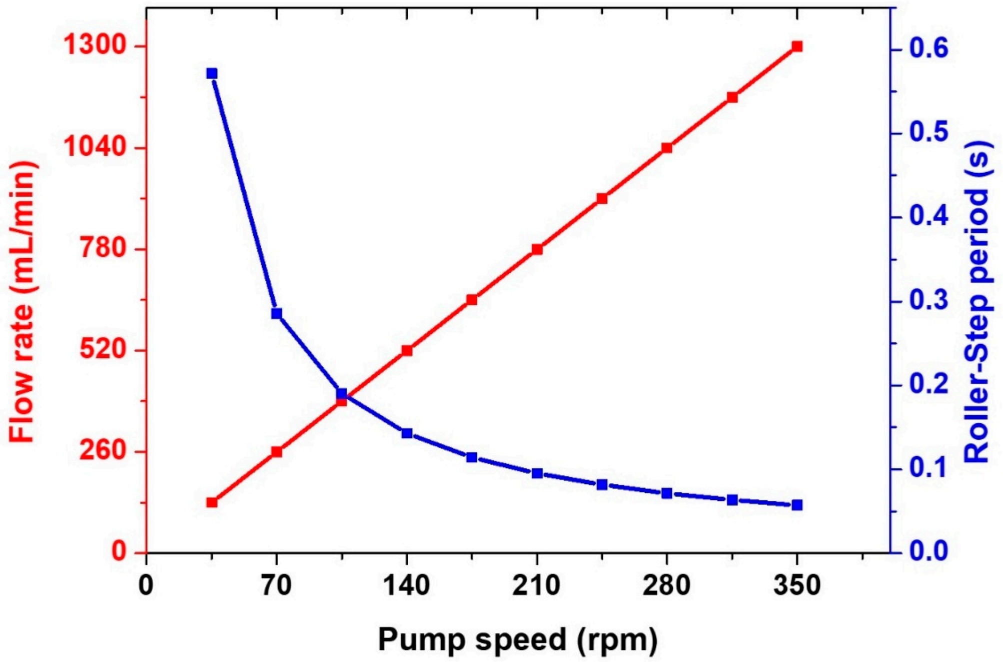

Figure S1. Characteristics of the peristaltic pump with 8 mm-cell FL-TENG.

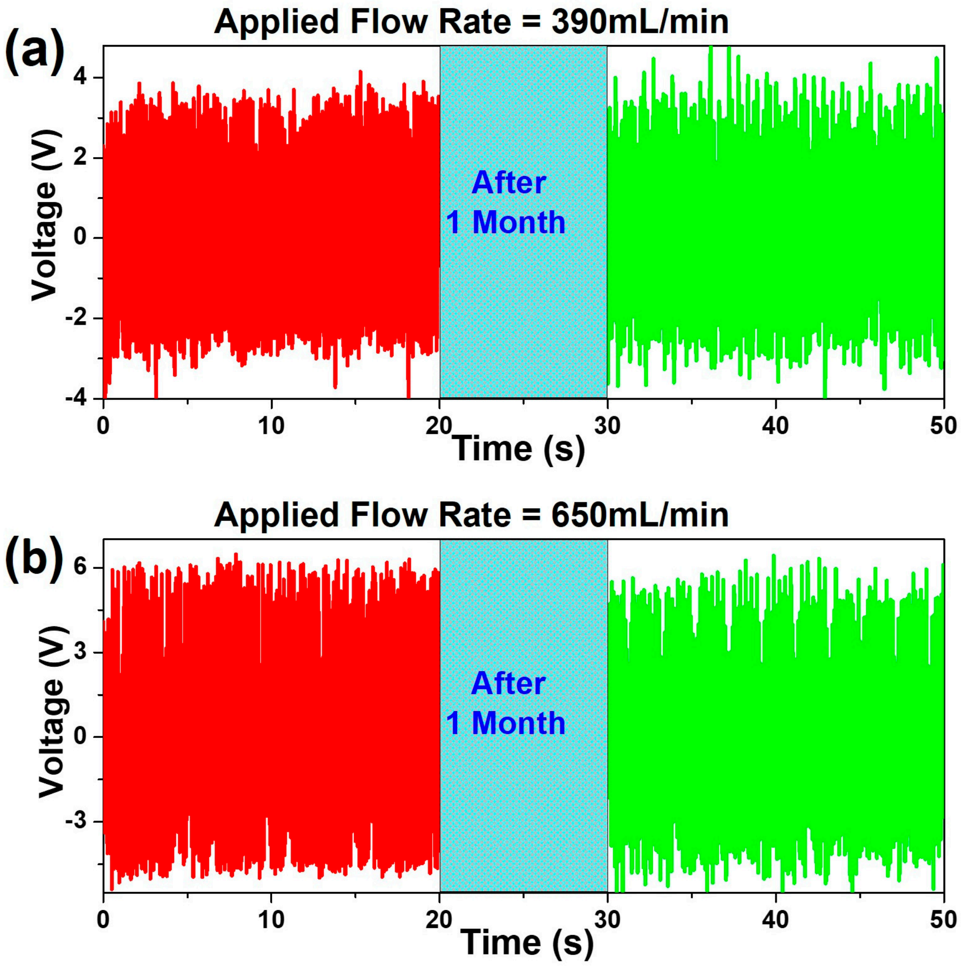

Figure S2. Stability and durability of the FL-TENG: output voltage measured at (**a**) 390 and (**b**) 650 mL/min of the FL-TENG at two moments one month apart.
